# Effects of *Bacillus amyloliquefaciens* FZB42 on Lettuce Growth and Health under Pathogen Pressure and Its Impact on the Rhizosphere Bacterial Community

**DOI:** 10.1371/journal.pone.0068818

**Published:** 2013-07-23

**Authors:** Soumitra Paul Chowdhury, Kristin Dietel, Manuela Rändler, Michael Schmid, Helmut Junge, Rainer Borriss, Anton Hartmann, Rita Grosch

**Affiliations:** 1 Research Unit Microbe-Plant Interactions, Helmholtz Zentrum München, German Research Center for Environmental Health (GmbH), Neuherberg, Germany; 2 ABiTEP GmbH, Berlin, Germany; 3 Leibniz Institute of Vegetable and Ornamental Crops, Großbeeren, Germany; Wageningen University and Research Centre, The Netherlands

## Abstract

The soil-borne pathogen *Rhizoctonia solani* is responsible for crop losses on a wide range of important crops worldwide. The lack of effective control strategies and the increasing demand for organically grown food has stimulated research on biological control. The aim of the present study was to evaluate the rhizosphere competence of the commercially available inoculant *Bacillus amyloliquefaciens* FZB42 on lettuce growth and health together with its impact on the indigenous rhizosphere bacterial community in field and pot experiments. Results of both experiments demonstrated that FZB42 is able to effectively colonize the rhizosphere (7.45 to 6.61 Log _10_ CFU g^−1^ root dry mass) within the growth period of lettuce in the field. The disease severity (DS) of bottom rot on lettuce was significantly reduced from severe symptoms with DS category 5 to slight symptom expression with DS category 3 on average through treatment of young plants with FZB42 before and after planting. The 16S rRNA gene based fingerprinting method terminal restriction fragment length polymorphism (T-RFLP) showed that the treatment with FZB42 did not have a major impact on the indigenous rhizosphere bacterial community. However, the bacterial community showed a clear temporal shift. The results also indicated that the pathogen *R. solani* AG1-IB affects the rhizosphere microbial community after inoculation. Thus, we revealed that the inoculant FZB42 could establish itself successfully in the rhizosphere without showing any durable effect on the rhizosphere bacterial community.

## Introduction

Soil-borne plant pathogens like *Rhizoctonia solani* Kühn [anamorph, teleomorph: *Thanatephorus cucumeris* (Frank) Donk] are difficult to control [1], [2]. Members of this species can survive as sclerotia for long periods in the soil. These persistent survival structures are considered as an important source of primary inoculum. The pathogen *R. solani* is responsible for crop losses on a wide range of economically important plant species worldwide [1]. The pre-plant fumigation with methyl bromide (MeBr) was the most effective way to control *R. solani* in the past by killing the sclerotia in the soil. This fumigant has however been banned as MeBr depletes the stratospheric ozone layer (classified as a Class I ozone-depleting substance; EPA, 2006). The complete ban of MeBr and the lack of other effective specific fungicides as well as the increasing demand by consumers for food that is less contaminated with pesticide residues have stimulated research for alternatives to combat soil-borne pathogens by means of biological control [2]. Among the control alternatives to the use of chemicals, the application of beneficial bacteria living in association with plant roots is promising [3]. Several studies have documented improved plant traits by interaction with plant associated bacteria, such as increased tolerance to biotic and abiotic stress, enhanced yield and increased biomass [4], [5], [6], [7].

Bacteria belonging to the Gram-positive genus *Bacillus* are ubiquitous in soil [8]. *Bacillus* species are attractive for use in farming systems because of their ability to form stable endospores which can survive the preparation of bacterial formulations [9]. Moreover, the ability to form spores is advantageous for storage, thereby increasing the shelf life [10]. Biocontrol activity has been demonstrated by strains of *Bacillus cereus* UW85 shown to suppress several plant diseases through the production of novel antibiotic Zwittermicin A [11]. *Bacillus amyloliquefaciens* FZB42 is the type strain for a group of plant-associated *Bacillus* spp. classified as *B. amyloliquefaciens* subsp. *plantarum* [12]. The genome of the strain was sequenced and the analysis showed that FZB42 is a bacterium with impressive capacity to produce metabolites with antimicrobial activity [13]. Its antifungal activity is due to non-ribosomal synthesis of the cyclic lipopeptides bacillomycin D and fengycin [14] whilst its antibacterial activity is mainly due to non-ribosomally synthesized polyketides [15]. Its plant colonizing ability was demonstrated with a GFP-labelled FZB42 strain on maize and *Arabidopsis* using confocal laser scanning microscopy [16]. Beneficial effects on plant growth and disease suppression were documented for *B. amyloliquefaciens* FZB42 on tomato, cucumber and cotton for example [17]–[21].

The genetic group *R. solani* AG1-IB is the causal agent of bottom rot on lettuce and occurs wherever lettuce is grown [22]. Although *Bacillus amyloliquefaciens* FZB42 is a commercially available product (RhizoVital®42, ABiTEP GmbH, Berlin, Germany), this is the first study addressing its rhizosphere competence, impact on lettuce growth and health under the influence of pathogen pressure.

The ability to colonize the rhizosphere effectively is a key factor for successfully improving the plant health and suppression of plant pathogens [23], [24]. Poor root colonization and subsequent insufficient production of antimicrobial metabolites can be a reason for inconsistency in activity of bacterial inoculants in the field [25], [26]. We hypothesized that FZB42 is a successful root colonizer of lettuce and subsequently could improve plant growth and health due to its ability to produce an array of secondary metabolites. To demonstrate our hypothesis, we performed pot experiments with biotic pressure in the form of *R. solani* inoculation. This was followed by experiments in a naturally pathogen-infested field. To provide a uniform and enhanced pathogen pressure, one set of experiments was performed with an additional pathogen (*R. solani*) inoculation in the field. A spontaneous rifampicin-resistant mutant (FZB42-Rif) which could be easily re-isolated from the rhizosphere was used for evaluation of the colonization ability. In addition to the direct assessment of the rhizosphere competence, knowledge of the interaction of FZB42 with the indigenous microbial community in the rhizosphere can improve our understanding of its ecological consequences in the field. Therefore, the bacterial community structure was studied using the 16S rRNA gene-based fingerprinting method terminal restriction fragment length polymorphism (T-RFLP) at two time points during lettuce growth. The use of molecular DNA-based tools has improved our understanding of microbial inoculants’ influence on the indigenous rhizosphere community [6], [27], [28]. Although T-RFLP provides a simplified representation of the dominant members in microbial communities compared to analyses provided by high-throughput sequencing technologies, it is a cost-effective, reproducible and robust method which has been widely used in rapid and effective fingerprinting of soil samples [29]. Additionally, the effect of different modes of application of FZB42 on plant growth and health was studied with the aim of subsequently recommending the effective dosage and application mode to the users of the commercially available product. The efficacy of an inoculant strain can be improved by mode of application based on characteristics of the inoculant and the application mode [30].

## Materials and Methods

### Inoculants Used in this Study

This study evaluated the effect of *Bacillus amyloliquefaciens* FZB42 on lettuce growth and health. All experiments were performed with the product Rhizovital® 42 liquid (ABiTEP GmbH, Berlin, Germany) which contained vital spores of FZB42 and no further additives with effects on the pathogen and on lettuce plants. A spontaneous rifampicin-resistant mutant (FZB42-Rif) of the bacterial strain was generated as described recently [6] and also used as a formulated product comparable to the wild-type strain. The mutant strain was stored at −80°C in Luria–Bertani broth (ROTH, Germany) containing 20% glycerol supplemented with rifampicin (75 µg ml^−1^).

To rule out the possibility of the mutant FZB42-Rif might having generated multiple physiological defects, the growth rates of wild-type versus rifampicin-resistant mutant were compared in Luria Bertani Broth at 30°C and 200 rpm shaking in three independent experiments. Samples were taken during the exponential growth phase and the amount of cells/ml of each strain was determined by plating on agar plates. To confirm that the rifampicin resistance remains stable during a longer period without antibiotic pressure, cells of FZB42-Rif were cultivated in Luria Bertani Broth without antibiotics at 30°C and 200 rpm shaking in three independent experiments. Samples were taken in intervals corresponding to 5, 10 and 15 generations and cells were plated on agar plates with and without rifampicin. The CFUs of both were compared.

*Rhizoctonia solani* AG1-IB (isolate O1/1 and 7/3) was obtained from the strain collection of the Leibniz Institute of Vegetable and Ornamental Crops (Großbeeren, Germany) and maintained on barley kernels at -20°C. The strain O1/1 was previously isolated from diseased lettuce plants at the experimental field [31]. Hence, this strain belonged to the indigenous *R. solani* AG1-IB population of the experimental field and was used for additional pathogen inoculation.

### Design of Pot Experiments

Two separate pot experiments were designed, the first one to study the impact of FZB42 on lettuce growth in presence of *R. solani* (isolate 7/3). In the second experiment, the colonization ability of FZB42-Rif with respect to different spore numbers (10^6^, 10^7^, 10^8^ spores/ml) was investigated. Moreover, the effect of FZB42 and the pathogen *R. solani* on the microbial community was studied by T-RLFP analysis (description see below).

In both experiments lettuce seeds (cv. Tizian, Syngenta, Bad Salzuflen, Germany) were germinated at 18°C in a seedling tray (92 holes) filled with a non-sterile mixture of quartz sand and substrate [Fruhstorfer Einheitserde Typ P, Vechta, Germany; chemical analysis (mg per l): N = 120, P = 120, K = 170, Mg = 120, S = 100, KCl = 1, organic substance = 167, peat = 309; pH 5.9] at a 1∶1 ratio (v/v). The seedlings were further cultivated at 20/15°C until planting in growth chamber (York, Mannheim, Germany; 16 h/8 h day/night cycle, 500 µmol m^−2^ s^−1^, 60/80% relative humidity). Lettuce seedlings were planted at 2-leaf stage into pots (500 ml) filled with the same substrate/sand mixture mentioned above and cultivated at 22/15°C until harvest. In the first experiment, lettuce seedlings were planted in pots with *R. solani* (+*Rs*) inoculation. In this case the mixture of quartz sand and substrate was inoculated with ten *R. solani*-infested barley kernels and incubated at 25°C for 1 week until planting. In the second experiment, each plant was drenched with 20 ml spore solution of FZB42 (10^7^ spores/ml) or FZB42-Rif (10^6^, 10^7^, 10^8^ spores/ml) after planting. The pots were watered lightly each day to maintain the substrate moisture and fertilized weekly (0.2% Wuxal TOP N, Wilhelm Haug GmbH & Co. KG, Düsseldorf, Germany). Each treatment included five replicates with five plants per replicate arranged in a randomized block design. Lettuce plants do not form a head during this growth period in pot experiments and the assessment of DS was not possible as compared to the field trial. Previous experiments showed that the pathogen affects the lettuce growth [4]. Hence, the impact of the pathogen was assessed based on shoot dry mass (SDM) 4 weeks after planting.

### Design of Field Experiment

The field trial was carried out at the Institute of Vegetable and Ornamental Crops (Golzow, Germany, 52° 34′ N, 14° 30′ E) with alluvial loam (total N 112; P 32.3; K 17.4; and Mg 9.1 mg/100 g soil; pH 6.5), the typical soil type of the area. The field was naturally infected with the bottom rot pathogen *R. solani* AG1-IB (31). The experimental field was divided into beds of 6.75 m^2^ with 11 plants per m^2^. Lettuce seedlings (cv. Tizian) were grown in peat blocks in a greenhouse at 20/15°C (16 h/8 h, day/night cycle), and planted at 3-4-leaf stage by hand in beds in five rows with intra-row and inter-row distances of 30 cm each (75 plants per bed). Each treatment included six replications arranged randomly. Sprinkler irrigation was applied after planting and during the vegetation of lettuce of 6 to 7 weeks [32] according to the irrigation schedule controlled by the computer program ‘BEREST’. Calculated soil water content and the expected evapotranspiration and precipitation of the next 5 days were the basis of irrigation decisions. A local weather station located 50 m away measured and recorded the air temperature. Within the first 3 weeks weeds were removed by hand. Fertilizer was added based on a chemical analysis of the soil and was applied 1 day before planting (N 100 kg/ha and K 120 kg/ha).

At harvest the SDM and disease severity (DS) were assessed from 18 plants per bed. DS was rated in four categories: 1 - healthy plants without distinct symptoms; 3 - symptoms only on first lower leaves in direct contact with the soil, light brown to dark brown spots, primarily on the underside of leaf midribs; 5 - brown spots on leaf midribs on lower and next upper leaf layer, rotting midribs and leaf blades; and 7 - severe disease symptoms on upper leaf layers, beginning of head rot to total head rot [33]. The arithmetic mean was calculated based on assessed DS of the individual plants.

The effect of *B. amyloliquefaciens* FZB42 and FZB42-Rif on lettuce growth, DS of bottom rot and microbial community in the rhizosphere of lettuce was studied under two different experimental conditions; i.e. naturally occurring pathogen pressure in the field and at higher pathogen pressure achieved by additional artificial pathogen inoculation (+*Rs*). In these treatments the plants were inoculated with barley kernels (one per plant) infested with *R. solani* (strain O1/1). The kernels were placed 1 cm deep at a distance of 2 cm from each lettuce plant at planting.

### Application Mode of FZB42 and FZB42-Rif in the Field Experiment

Lettuce plants were treated with FZB42 or FZB42-Rif at 2-3-leaf stage 1 week before planting into the field beds. Each seedling tray holding 150 plants was watered with 1.74 l spore suspension (10^7^ CFU ml^−1^) of FZB42 or FZB42-Rif respectively. Four days after planting, the lettuce plants at 3-4-leaf stage were treated with a spore suspension (10^7^ CFU ml^−1^) of FZB42 or FZB42-Rif. An amount of 0.5 l spore suspension was applied by hand sprayer to each bed.

The effect of two times application (TTA) before and after planting was evaluated in comparison to a single application (SA) after planting (without treatment of seedlings before planting) for FZB42 and the effect of TTA for FZB42-Rif. Additionally, the effect of a double concentration of FZB42 (TTA2x) was compared to the effect of single concentration of FZB42 (TTA).

### Root Colonization by the Inoculant FZB42-Rif

The survival and root colonization efficiency of FZB42-Rif was evaluated at three time points during the growth of lettuce in the field (at planting, 2 and 5 weeks after planting) and in the pot (at planting, 2 and 4 weeks after planting) experiments.

For each sampling time, three plants (field experiment) or one plant (pot experiment) of four replicates respectively were collected. Loosely adhering soil was removed from the roots and 5 g of lateral roots and tap-roots with rhizosphere soil were suspended in 10 ml sterile saline (0.3% NaCl) respectively and shaken vigorously in 100 ml Erlenmeyer flasks containing 15 glass beads (0.6 mm in diameter) on a rotary shaker for 1 h at 307 rpm. The surviving total cell number of FZB42-Rif was determined by plating serial dilutions of the rhizosphere suspension on nutrient agar I (TN 1164, SIFIN GmbH Berlin, Germany) with addition of rifampicin (75 µg ml^−1^) and cycloheximide (100 µg ml^−1^). The Petri dishes were incubated at 28°C for 2 to 3 days before CFUs were counted. Simultaneously, aliquots of the rhizosphere suspension were heated at 90°C for 30 min to destroy the vegetative cells and determine the number of FZB42-Rif spores. The numbers of vegetative cells were calculated based on the assessed total cell and spore numbers. All isolated colonies were checked for the typical morphology of FZB42. To prove the authenticity of re-isolated colonies on selective agar plates, chromosomal DNA was isolated from different morphologically typical colonies and PCR was performed with specific primers for FZB42 as previously described [12]. For analysis of the microbial rhizosphere community, the remaining rhizosphere suspension was centrifuged at 13, 000 *g* for 5 min. The supernatant was discarded and the cell pellet stored frozen at −20°C. Cell pellets of the other treatments were prepared in the same way for analysis of the indigenous microbial rhizosphere community.

### DNA Extraction, PCR Amplification, and T-RFLP Analysis

The total community DNA of the bacterial fraction was extracted from the frozen rhizosphere pellet. Approximately 500 mg fresh weight root material was transferred into 2 ml screw-cap vials that contained 1 g of a mixture of ceramic and silica particles. DNA extractions were performed using the Fast DNA® SPIN Kit for Soil (MP Biomedicals, Eschwege, Germany) according to the manufacturer’s instructions. After measurement of the DNA concentration using a NanoDrop spectrophotometer (ND-1000, NanoDrop Technologies, Wilmington, USA), the DNA extracts were stored at −20°C.

For T-RFLP analysis of bacterial communities partial 16S rRNA genes were amplified using the primers 27f-FAM (AGAGTTTGATCMTGGCTCAG) [34] and 907r (CCGTCAATTCCT TTGAGTTT) [35]. The reaction mixtures (50 µl) contained 10 ng of extracted DNA as a template and 1X AmpliTaq Gold® PCR Master Mix (Applied Biosystems). A touch-down amplification protocol was followed, which included the following reactions: initial denaturation at 95°C for 5 min, 10 cycles of denaturation at 95°C for 1 min, annealing at 65°C -55°C for 1 min, elongation at 72°C for 1 min followed by 20 cycles of denaturation at 95°C for 1 min, annealing at 55°C for 1 min, elongation at 72°C for 1 min and a final extension at 72°C for 10 min. Polymerase chain reaction (PCR) products were purified using the QIAquick PCR Purification Kit (Qiagen GmbH, Hilden, Germany) as recommended by the manufacturer. The concentrations of DNA fragments were determined using the NanoDrop spectrophotometer.

Approximately 100 ng of purified PCR product was digested overnight, at 37°C with 5 U of MspI in recommended 1X Buffer Tango™ (Fermentas GmbH). Following digestion, samples were desalted on SigmaSpin™Post-Reaction Clean-up Columns (Sigma, Germany); aliquots of 3 µl were mixed with 10 µl of HiDi™ Formamide (Applied Biosystems) and 0.3 ml of the internal DNA standard MapMarker®1000 (BioVentures). The electrophoretic separation of desalted digests was performed on an automated DNA sequencer (ABI 3730, Applied Biosystems, Applera Deutschland GmbH, Darmstadt, Germany) and the lengths of terminal restriction fragments (T-RFs) were determined using GeneMapper® v3.5 software (Applied Biosystems). Signals with a peak height of more than 100 fluorescence units were included in further analyses [36].

### T-RFLP Data Processing

Raw data from GeneMapper™ were exported to T-REX, an online software for the processing and analysis of T-RFLP data (http://trex.biohpc.org/) [37]. The T-RFLP data were subjected to several quality control procedures: noise filtering (peak area, standard deviation multiplier = 1), T-RF alignment (clustering threshold = 1 bp), only T-RFs between 50 and 500 bp were included in the analyses. The analysis of data matrices made use of the additive main effect and multiplicative interaction (AMMI) model based on the analysis of variance (ANOVA) [38]. The relative abundance of a detected T-RF within a given T-RFLP profile was calculated as the respective signal height of each peak divided by the total peak height of all the peaks of the T-RFLP profile. The T-REX-constructed data matrices were then exported to PAST [39]. Nonmetric multidimensional scaling (NMS) using Bray-Curtis distance measure was also performed in PAST to analyze the data. The AMMI ordination results were graphed as scatter plot provided by T-REX and Microsoft Excel.

### Statistical Analysis

The data analysis of shoot dry mass (SDM), disease severity (DS) and inoculant densities were performed with the STATISTICA program (StatSoft Inc., Tulsa, Ok USA). The SDM and inoculant densities data were analyzed using ANOVA with Dunnett’s test procedure with *P* = 0.1 and the median of DS using nonparametric statistics with the Kruskal Wallis test with *P*≤0.05. The determined cell densities of FZB42-Rif were logarithmically (Log _10_) transformed before a three-way ANOVA analysis according to Tukey HSD test procedure with *P* = 0.1.

To test the significant differences between T-RFLP groups obtained with T-REX, the data matrices were then exported to PAST [39]. A one-way ANOSIM [40] was performed to reveal R values. The R statistics measure whether a separation of community structure is found (R = 1), or whether no separation occurs (R = 0). Values above 0.75 are interpreted as well-separated, above 0.5 as separated but overlapping and R less than 0.25 as barely separable [41]. The P-values were corrected with the Bonferroni correction to reveal significant differences [42].

## Results

### The Effect of FZB42 and FZB42-Rif on Lettuce Growth and Root Colonization in Pot Experiments

A significantly lower lettuce dry mass (2.62 g per plant) was observed under biotic stress with *R. solani* in comparison to the dry mass of the untreated control (6.48 g per plant). A comparable dry mass (6.39 g per plant) in the treatment with FZB42 and pathogen inoculation to the SDM in the untreated control (6.48 g per plant) was observed. Hence, the application of FZB42 to lettuce seedlings could significantly limit the effect of the pathogen.

Lettuce growth was not affected by application of variable spore numbers (10^6^, 10^7^ and 10^8^ spores/ml) of FZB42-Rif in the second experiment. A SDM of 3.1 g per plant was measured in the treatment with 10^6^ spores/ml, compared to 3.5 g per plant in the treatment with 10^7^ spores/ml, and 3.6 g per plant in the treatment with 10^8^ spores/ml. The untreated control had a dry mass of 3.2 g per plant.

The total density (CFU) of FZB42-Rif in the lettuce rhizosphere at all sampling times corresponded to the applied spore numbers at planting and decreased significantly in all treatments (10^6^, 10^7^ and 10^8^ spores/ml) within 4 weeks (Table 1). When comparing the spores versus vegetative cells found 2 and 4 weeks after planting, the spore density of FZB42-Rif decreased significantly in all treatments whereas the density of vegetative cells was not affected in any of the treatments within the 4 weeks.

**Table 1:** Colonization densities of *Bacillus amyloliquefaciens* FZB42-Rif on lettuce root in pot experiments in respect to applied spore numbers.

Applied spore density [spores/ml] and cell form	Log _10_ CFU g^−1^ root dry mass		
	at planting	at 2 weeks	at 4 weeks
FZB42-Rif 10^6^			
CFU	5.96 A	5.72 A	4.95A*
Spores	–	5.63 a	4.08 a*
Vegetative cells	–	4.88 a	4.75 a
FZB42-Rif 10^7^			
CFU	6.84 BC	6.01 A*	5.74 BC*
Spores	–	5.82 a	5.27 a*
Vegetative cells	–	5.55 a	5.51 a
FZB42-Rif 10^8^			
CFU	7.23 C	6.84 B*	6.41C*
Spores	–	6.59 a	5.10 a*
Vegetative cells	–	6.47 a	6.38 a

Three-way ANOVA according to Tukey HSD test revealed that the time (*P* = 1.28 10^−6^) and the applied spore numbers (*P* = 8.18 10^−9^) significantly affected the total density (CFU) of FZB42-Rif in the rhizosphere of lettuce. Time had a significant influence on the density of cells (*P* = 0.001025). Different capital letters denote significant differences between total densities (CFU) of FZB42-Rif depending on applied spore numbers (10^6^, 10^7^, 10^8^ spores/ml) per column and different small letters between densities of spores and vegetative cells per column (at the same time point). The asterisk denote significant differences in density of FZB42-Rif compared to density at planting (CFU) or to density 2 weeks after planting (spores, vegetative cells).

### The Effect of FZB42 and FZB42-Rif on Lettuce Growth and Disease Severity Of Bottom Rot in the Field

A total of 108 plants per treatment (six replicates and 18 plants per replicate) were used to evaluate the effect of the application mode of FZB42 under the two different experimental conditions, natural and higher pathogen pressure, on lettuce growth and DS after a cultivation time of 6 weeks. No significant effect of additional *R. solani* inoculation (*P* = 0.392) on SDM and DS was found based on three way ANOVA (Table 2). A lettuce SDM of 34.3 g per plant on average of all treatments under natural pathogen pressure was measured compared to 31.9 g per plant with additional pathogen inoculation. The application mode of FZB42 significantly affected the lettuce growth and DS of bottom rot (*P* = 0.0473). A two times application with FZB42 (TTA and TTA2x) and FZB42-Rif (TTA) before and after planting resulted in improved lettuce SDM and decreased DS of bottom rot whereas a single application after planting (SA) did not (Table 2). No different effects on lettuce growth and DS of bottom rot were observed when comparing the treatments with FZB42 (TTA) and FZB42-Rif (TTA).

**Table 2:** Effects of *Bacillus amyloliquefaciens* FZB42 and FZB42-Rif on lettuce growth at two different experimental conditions: natural pathogen pressure and additional inoculation of *Rhizoctonia solani* AG1-IB (*+R*s) in the field.

Treatment	Applica-tion mode	SDM [g/plant]	DS
Control	–	26.5	5.0
FZB42	TTA	38.9*	3.0*
FZB42	SA	32.1	3.0*
FZB42	TTA2x	37.2*	3.0*
FZB42-Rif	TTA	36.8*	3.0*
Control +*Rs*	–	26.4	5.0
FZB42+*Rs*	TTA	33.0	3.3*
FZB42+*Rs*	SA	29.9	4.7
FZB42+*Rs*	TTA2x	34.5*	4.0
FZB42-Rif +*Rs*	TTA	37.0*	3.0*

Evaluation of the lettuce shoot dry mass (SDM) and disease severity (DS). Numbers followed by an asterisk in one column are significantly different from the corresponding control according to Dunnett’s test procedure *P* = 0.1 for SDM and according to Kruskal Wallis test *P*≤0.1 for DS.

TTA – two times application 1 week before planting and 4 days after planting.

SA – Single application 2 days after planting.

TTA2x –two times application with double concentration.

### Root Colonization by the Inoculant FZB42-Rif in the Field

No significant difference was observed in the growth rate of the FZB42-Rif mutant and the wild type in Luria Bertani Broth. A generation time of 48.1±0.49 min was assessed for FZB42 and of 48.1±2.6 min for rifampicin resistant mutant. Plating of FZB42-Rif cells on agar plates after 5, 10 and 15 generations of growth in Luria Bertani Broth without antibiotic pressure proved that the rifampicin resistance remains stable during a longer period without antibiotic pressure.

The lettuce rhizosphere, including lateral as well as tap-roots, was actively colonized by FZB42-Rif during the growth period of lettuce in the field (Table 3). Three-way ANOVA revealed that the total density of FZB42-Rif decreased significantly from 7.45 to 6.51 Log _10_ CFU g^−1^ root dry mass (rdm) on average (*P*<0.001) in the rhizosphere of lettuce within 5 weeks. A significantly higher density of spores (6.73 Log _10_ g^−1^ rdm) compared to vegetative cells (6.18 Log _10_ g^−1^ rdm) was assessed at both time points after planting (*P*<0.0001). Moreover, additional inoculation of *R. solani* has a significant influence on the density of FZB42-Rif in the rhizosphere (6.63 without and 6.51 Log _10_ CFU g^−1^ rdm with *R. solani* inoculation on average of densities 2 and 5 weeks after planting, *P* = 0.02). The spore numbers of FZB42-Rif decreased significantly within the growth period of lettuce whereas the density of vegetative cells decreased only in the treatment with additional *R. solani* inoculation. Significant differences in density of vegetative cells and spores of FZB42-Rif were found at each sampling time except the treatment without additional pathogen inoculation 5 weeks after planting (Table 3). To make sure that only FZB42-Rif colonies were taken into account all colonies were checked for their typical FZB42 morphology. 25 of these colonies were tested further for their authenticity by PCR with specific primers. All of these colonies obtained the expected bar-code band of 785 bp [12].

**Table 3:** Root colonization by *Bacillus amyloliquefaciens* FZB42-Rif in the field.

Cell form	Lateral root: Log _10_ CFU g^−1^ root dry mass		
	at planting^a^	at 2 weeks	at 5 weeks
FZB42-Rif			
CFU	7.45 a	7.22 a	6.61 b
Spores	7.25 a A	7.13 a A	6.44 b A
Vegetative cells	7.02 a A	6.48 b B	6.10 b A
FZB42-Rif +*Rs*			
CFU	–	7.17 b	6.41c
Spores	–	7.05 a A	6.30 b A
Vegetative cells	–	6.44 b B	5.69 c B
	**Tap-root: Log _10_ CFU g** ^−**1**^ ** root dry mass**		
FZB42-Rif +*Rs*			
CFU	–	6.09 a	5.15 b
Spores	–	6.09 a A	5. 01 b A
Vegetative cells	–	5.50 a B	4.44 b B

Colonization density of FZB42-Rif on lateral and tap-roots at planting, 2 and 5 weeks after planting under the two different management practices, natural and higher pathogen pressure (*+R*s). Three-way ANOVA according to Tukey HSD test revealed that the time (*P* = 2.16 10^−11^) significantly affected the density of FZB42-Rif in the rhizosphere of lettuce and that significant differences (*P* = 3.45 10^−10^) in density between spores and vegetative cells exist.

a The data represents the density of FZB-Rif at planting time, hence are the same for both treatments FZB42-Rif and FZB42-Rif+*Rs*.

Density followed by the same small letters denote no significant differences between densities at various time points (per row) and capital letters between densities of spores and vegetative cells at the same time point (per column).

### The Effect of FZB42-Rif and *R. solani* on the Rhizosphere Bacterial Community in Pot Experiments

**Figure 1:**
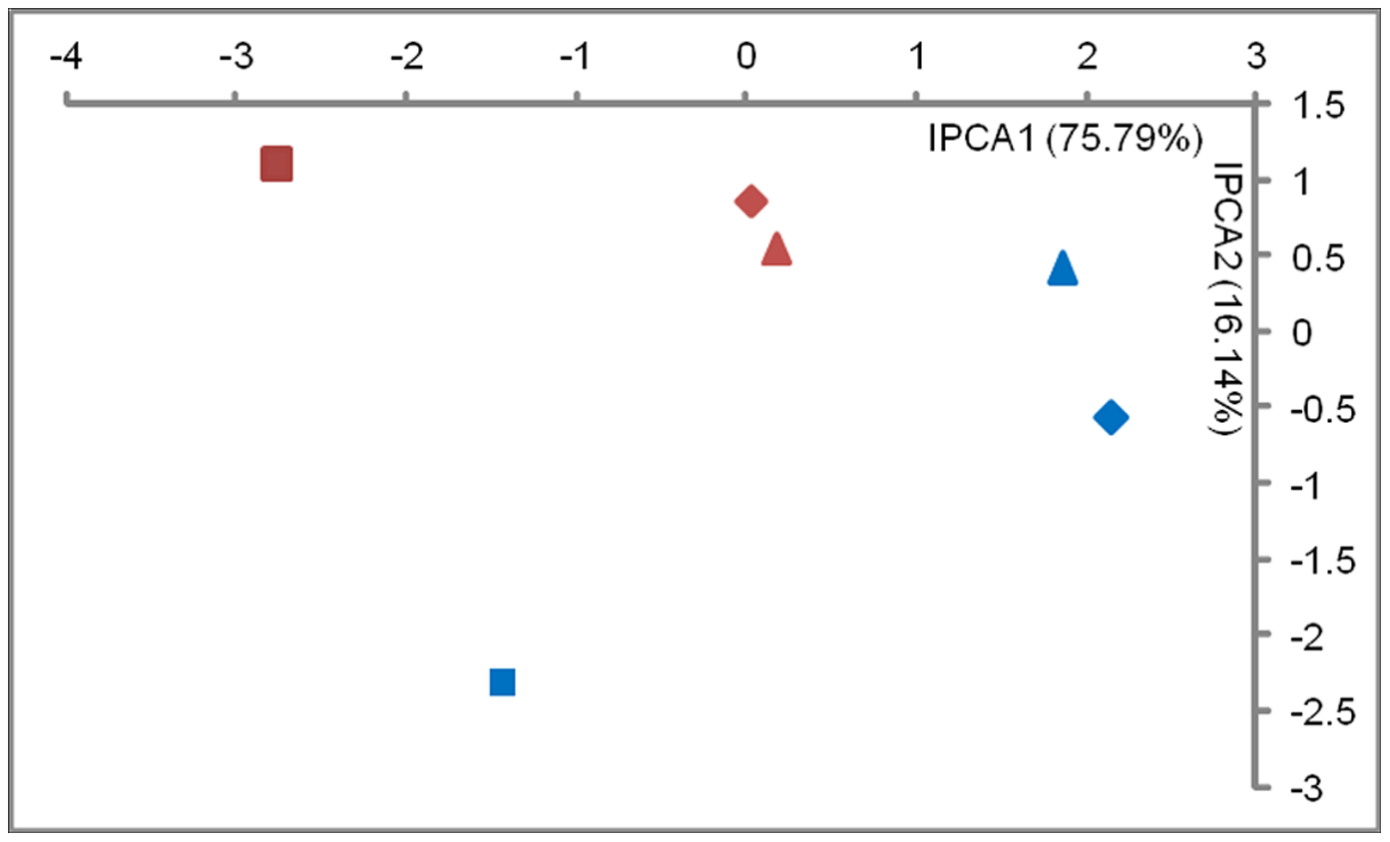
Effect of *Bacillus amyloliquefaciens* FZB42-Rif application and *Rhizoctonia solani* AG1-IB inoculation on the rhizosphere bacterial community in pot experiments. Ordination plot of bacterial community T-RFLP data analyzed with AMMI. Symbols ▴ represent plants treated with FZB42-Rif; ♦ uninoculated controls; ▪ plants grown in presence of *R. solani* (+*Rs*) Red: Sampling after 2 weeks of growth; Blue: Sampling after 4 weeks of growth.

As described above, we found no significant differences in lettuce growth due to application of variable spore numbers of FZB42-Rif. Therefore, we selected only the treatment with 10^7^spores/ml for T-RFLP analysis. Rhizosphere samples collected from inoculated plants, 2 weeks and 4 weeks after inoculation were compared with non-inoculated control plants and plants grown in the presence of the fungus *R. solani* (+*Rs*). The microbial community pattern generated by T-RFLP is grouped into environments as revealed by the ordination results of the AMMI (Figure 1). Analysis of the datasets showed that the inoculation of FZB42-Rif was not a major source of variation in the pattern, whereas the presence of the *R. solani* was the major driver as captured by the first interaction principal component (IPCA1). The sampling time was the secondary driver of the community structure, separating on IPCA2.

### The Effect of FZB42 on the Rhizosphere Bacterial Community in the Field

**Figure 2:**
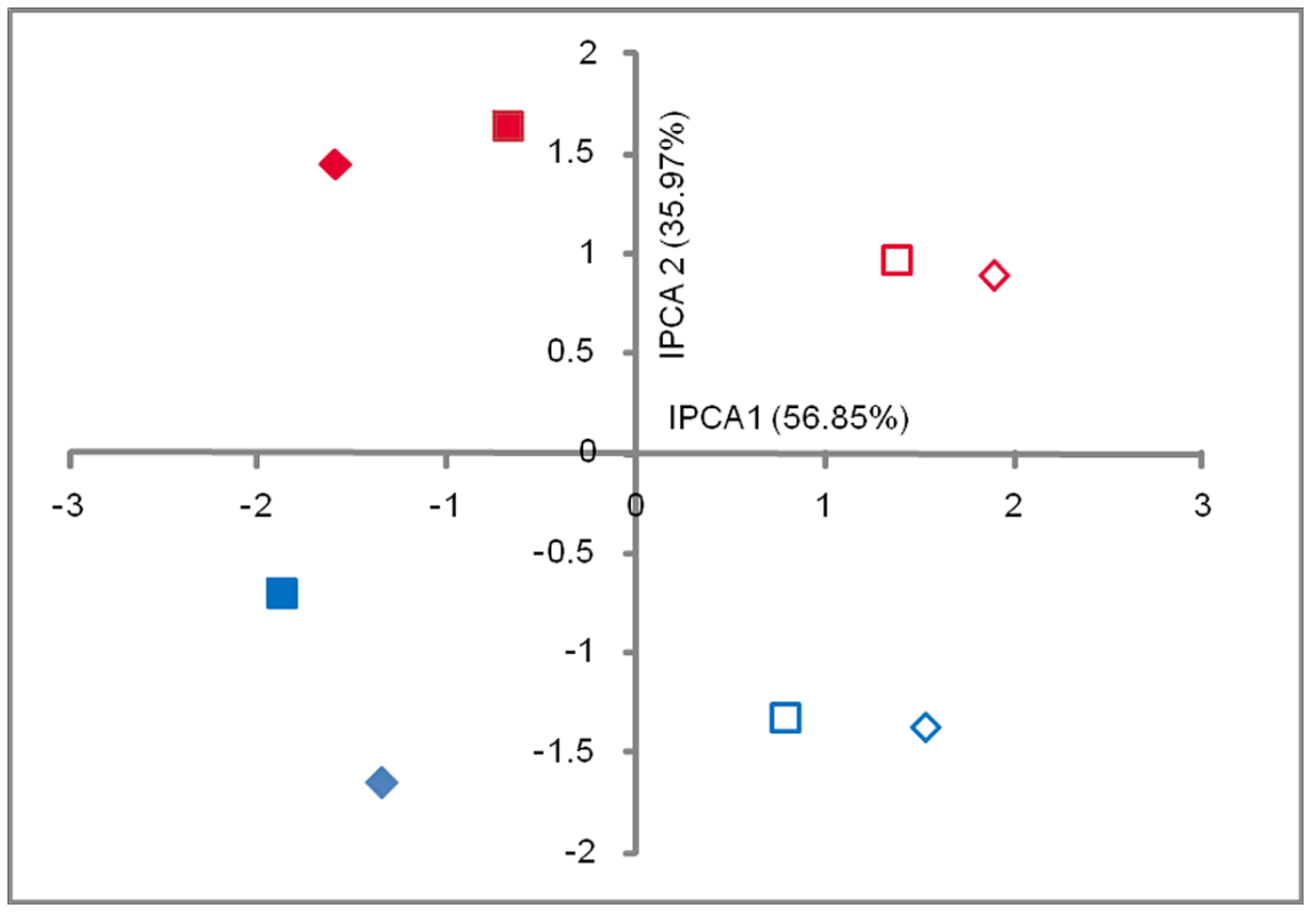
Effect of *Bacillus amyloliquefaciens* FZB42 application on the rhizosphere bacterial community in the field. Ordination plot of bacterial community T-RFLP data analyzed with AMMI. Symbols ♦ represent plants treated with FZB42; ▪ uninoculated controls. Open data points represent plants with natural pathogen pressure of *Rhizoctonia solani* in the field and closed data points at additional pathogen inoculation of *R. solani* AG1-IB. Red: Sampling after 2 weeks of growth; Blue: Sampling after 5 weeks of growth.

Plants inoculated with FZB42 versus non-inoculated controls were studied under two different experimental conditions, namely under natural pathogen pressure and with additional pathogen inoculation. Samples were collected from the field 2 weeks and 5 weeks after planting. The T-RFLP groups, referred to as Environments as revealed by the ordination results of the AMMI (Figure 2), showed that the T-RFs of the rhizosphere bacterial community responded differently to the experimental conditions as well as to time points. Each environment in the T-REX program consisted of 12 T-RFLP patterns (4 plant samples with 3 technical replicates of each). Analysis of the datasets revealed that additional pathogen inoculation (different experimental conditions) was the major driver as captured by the first interaction principal component analysis (IPCA1). The sampling time was the secondary driver of the community structure, separating on IPCA2. There was no major effect of the inoculation of FZB42 on the rhizosphere bacterial community because there was no major source of variation apparent in the pattern.

To examine if non-parametric analyses may yield more discriminatory ordination results, we performed NMS analysis. The ordination produced was very similar to the eigenvector-based method evaluated above (data not shown). The ordination plot clearly distinguished differences in the communities based on experimental conditions and time points. However, the samples from FZB42 application clustered closely with the samples from non-inoculated controls.

**Figure 3:**
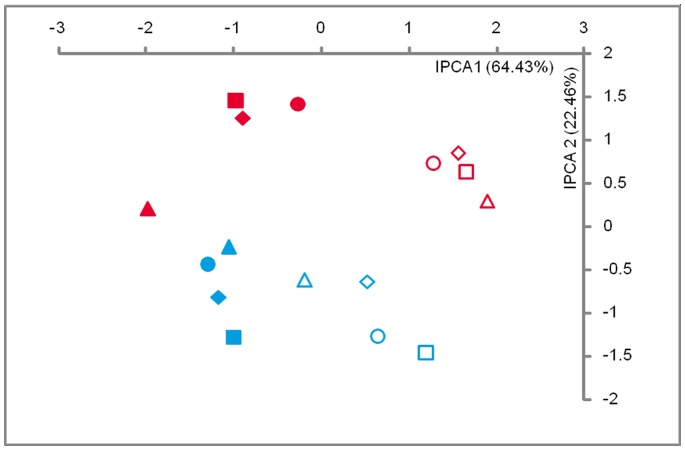
Effect of application mode of *Bacillus amyloliquefaciens* FZB42 on the rhizosphere bacterial community in the field. Ordination plot of T-RFLP data analyzed with AMMI showing effect of FZB42 on bacterial community under consideration of the application mode. Symbols: • uninoculated controls; ▪ TTA – two times application 1 week before planting and 4 days after planting; ♦ SA – single application 2 days after planting;▴TTA2x – two times application with double concentration. Open data points represent plants with natural pathogen pressure of *Rhizoctonia solani* in the field and closed data points at additional pathogen inoculation of *R. solani*. Red: Sampling after 2 weeks of growth; Blue: Sampling after 5 weeks of growth.

The data were further analyzed to test if the mode of application of FZB42 had any significant effect on the rhizosphere microbial community (Figure 3). The experimental conditions (natural pathogen pressure and additional pathogen inoculation of *R. solani*) were the primary driver of the community structure and that the sampling time was the secondary driver (as revealed by the IPCs). Although some minor differences were observed in case of different modes of application, they did not show a driving effect on the distribution of the T-RFs in the observed community pattern.

Analysis of sources of variation of the dataset revealed that the bacterial communities had a relatively low interaction (Table 4) composed of 14.15% pattern and 1.38% noise. Analysis of similarity between the different communities was performed by using ANOSIM and the resulting R values are depicted in Table S1. From these values, it is apparent that the communities changed with time and due to the artificial pathogen application. The shift in the bacterial community was statistically significant as verified by the ANOSIM analysis. This was the case of non-inoculated controls as well as of all modes of applications of FZB42. However, the values of R were close to 0 when inoculated samples were compared with non-inoculated controls for each set (Table S1), indicating barely separable differences in the community structure.

**Table 4:** Percent variation in the bacterial community T-RFLP data revealed by online software T REX.

Source	FZB42 vs. control	Different modes of application
Main Effects		
T-RFs	84.47	84.65
Environments	0	0
Interaction Effects	15.53	15.35
Pattern	14.15	13.71
Noise	1.38	1.64
Sample Heterogeneity	1.1	1.5

## Discussion

In this study we used a combination of pot and field investigations to study the rhizosphere competence of *B. amyloliquefaciens* FZB42 and its impact on lettuce growth and health in presence and absence of the pathogen *R. solani* AG1-IB, and the impact on the indigenous rhizosphere microbial community. Moreover, the effects of different application modes of FZB42 were studied in the field.

Both results of pot and field experiments demonstrated that FZB42 is able to effectively reduce the disease severity of bottom rot caused by *R. solani* AG1-IB on lettuce. The spontaneous rifampicin resistant mutant FZB42-Rif used for colonization studies showed comparable effects on lettuce growth and health as the wild type FZB42. Supporting our hypothesis, the results illustrated a sufficient rhizosphere colonization density of FZB42-Rif of approximately 6.3 to 7.0 Log _10_ CFU g^−1^ root dry mass in both the pot and the field during the whole growth period of lettuce. But the density was affected by the applied FZB42 spore number and both spores and vegetative cells were present in the rhizosphere. However, in absence of the pathogen, lettuce growth was independent of the applied cell number in the pot experiment. Interestingly, our studies indicated that the density of vegetative cells did not decrease during the growth period of lettuce in absence of the pathogen (pot experiment) and in the treatment without additional pathogen inoculation in the field. In the set of experiments performed with an additional pathogen inoculation in the field it can be assumed that there was a higher pathogen density. Hence, a competition of the vegetative cells and the pathogen for root exudates could be a plausible explanation for the decrease in the number of vegetative cells.

Moreover, our studies demonstrated that the application mode is important for efficacy of the biocontrol effect exerted by FZB42. An effective suppression of *R. solani* was found only after two times application of FZB42 before and after planting (TTA) and this effect was not found to improve significantly by application of higher spore numbers (TTA2x). For the settlement of the inoculated strain in the rhizosphere in a sufficient high number, it might be important that the microflora in the rhizosphere of young plants is not yet stabilized [43]. Lugtenberg and Kamilova [23] suggested that a high rhizosphere competence of an inoculant strain is a key factor for successful disease control. There are several possible mechanisms of pathogen control, like the production of antifungal metabolites. *B. amyloliquefaciens* FZB42 can produce more than a few secondary metabolites with antifungal and antibacterial activities that apparently enable the bacterium to colonize successfully the highly competitive rhizosphere habitat. Until now, it has not been completely elucidated to what extent secondary metabolites are produced *in situ*. Recent studies have implied that surfactin production by FZB42 may be most important in colonizing plant roots [16]. However, in another study with *B. amyloliquefaciens* S499, it has been shown that the antifungal lipopeptides fengycin and iturin A are poorly expressed *in planta* [44]. The elicitation of induced systemic resistance (ISR) can also be a potential mechanism of FZB42 to reduce severity of bottom rot. This was demonstrated by several *Bacillus* spp. strains that suppressed diseases on various hosts [45]. However, further information from our ongoing studies about the metabolome of FZB42 may further reveal the actual mechanisms which are involved in pathogen control by FZB42.

Although specific microorganisms are able to protect plants against soil-borne pathogens, their efficacy is largely influenced by their interactions with the indigenous microbial community in the rhizosphere [43]. Therefore, another interest was to study how the rhizosphere bacterial community changes in response to the application of *B. amyloliquefaciens* FZB42. Our analysis revealed differences in responses of T-RFs (bacterial population) to imposed conditions and environments. Hence the TxE interaction effect as calculated with the AMMI in T-REX is of primary interest [38]. The interaction effects detected in this study (Table 4) were relatively low, which means that the inoculant had no pronounced effect on the rhizosphere bacterial communities. The relation between the variation and the origin of the samples has been previously described [38] and as expected, the variation was low in this case as all the samples were collected from the same field reflecting a short length of ecological gradient. This is also supported by the relatively low beta (β) diversity, which is the variation in species composition among sites in the geographic area of interest (Table 4) [46]. Soil-based T-RFLP data has been described as having short gradients and generally not being very complex compared to other types of ecological community data [38], [47]. Although the interaction effects were low, the ordination analyses (Figure 1, 2 and 3) could focus on the interaction and the interaction principal components (IPCs) account for nearly the entire interaction signal. Ordination graphs constructed with different methods did not deviate from each other and showed a low complexity of the T-RFLP data, which has been previously described in various ecological studies [38].

From our results it is apparent that the application of FZB42, independent of its mode of application, did not have a major impact on the rhizosphere bacterial community – as also shown for *B. amyloliquefaciens* BNM122 on soybean [48]. Previous studies with lettuce treated with inoculants like *Serratia plymuthica* 3Re4-18, *Pseudomonas trivialis* 3Re2-7, *P. fluorescens* L13-6-12 and *P. jessenii* RU47, also merely have a minor and transitory effect on the indigenous rhizosphere community; however they showed field site-specific and seasonal changes [6], [28], [49]. Our results revealed a clear shift in bacterial community structure over a time period of between 2 and 5 weeks after planting.

The results clearly indicate that the additional inoculation with *R. solani* AG1-IB showed an effect on the rhizosphere microbial community. This confirms our findings in the pot experiment (Figure 1) and corroborates previous observations. These studies demonstrated that under high pathogen pressure, *R. solani* affects populations of bacteria and fungi in the rhizosphere [6], [28], [50]. The interaction between the pathogen and the plant can result in changes in the root exudation pattern which influences the indigenous rhizosphere microbial community [51]. Moreover, alterations in rhizosphere composition upon infection might be affected by induced excretion of antimicrobial compounds by infected roots or through the recruitment of beneficial microbes. Some recent studies have indicated that when they need it, plants do indeed call for microbial help [43], [52], [53]. On the one hand, the application of the enhanced pathogen pressure may be an unnatural condition; but on the other, it simulates situations in which the plants are attacked by soil-borne pathogens. Therefore, such experiments can provide insight into the complex interactions occurring in the rhizosphere microbiome.

Confirming our hypothesis, this study indicates that the application of FZB42 on the field grown lettuce is effective in controlling the bottom rot disease not through community effects but via direct interaction or systemic resistance effects. It also shows that the inoculant FZB42 is a good colonizer of the rhizosphere without showing any measurable effects on the rhizosphere bacterial community. Although the T-RFLP analysis does not provide any information on the phylogeny of the rhizosphere bacteria, the observed temporal change in the community profile as well as its response to the presence of enhanced pathogen pressure was well reflected by the T-RFs and the interaction effects. Our ongoing research using next-generation sequencing techniques will reveal more information about the community composition and bring new insights into the interactions of *B. amyloliquefaciens* FZB42 and *R. solani* on the roots of field-grown lettuce plants.

## Supporting Information

Table S1 ***R*** **test statistic values.**
(DOCX)

## References

[1] González-García V, Portal Onco MA, Rubio Susan V (2006) Review. Biology and Systematics of the form genus *Rhizoctonia* . Span J Agric Res 4: 55–79.

[2] Martin FN (2003) Development of alternative strategies for management of soilborne pathogens currently controlled with methyl bromide. Ann Review Phytopathol 41: 325–350. 10.1146/annurev.phyto.41.052002.095514 14527332

[3] Adesemoye AO, Kloepper JW (2009) Plant-microbes interactions in enhanced fertilizer-use efficiency. Appl Microbiol Biotechnol 85: 1–12. 19707753 10.1007/s00253-009-2196-0

[4] Grosch R, Faltin F, Lottmann J, Kofoet A, Berg G (2005) Effectiveness of 3 antagonistic bacterial isolates to control *Rhizoctonia solani* Kühn on lettuce and potato. Can J Microbiol 51: 345–353. 15980897 10.1139/w05-002

[5] Ongena M, Duby F, Jourdan E, Beaudry T, Jadin V, et al (2005) *Bacillus subtilis* M4 decreases plant susceptibility toward fungal pathogens by increasing host resistance associated with differential gene expression. Appl Microb Cell Physiol 67: 692–698. 10.1007/s00253-004-1741-0 15578181

[6] Adesina MF, Grosch R, Lembke A, Vatchev TD, Smalla K (2009) In vitro antagonists of *Rhizoctonia solani* tested on lettuce: rhizosphere competence, biocontrol efficiency and rhizosphere microbial community response. FEMS Microbiol Ecol 69: 67–74. 10.1111/j.1574-6941.2009.00685.x 19486156

[7] Müller H, Westendorf C, Leitner E, Chernin L, Riedel K, et al (2009) Quorum-sensing effects in the antagonistic rhizosphere bacterium *Serratia plymuthica* HRO-C48. FEMS Microbiol Ecol 67: 468–478. 19220861 10.1111/j.1574-6941.2008.00635.x

[8] Hallmann J, Rodriguez-Kabana R, Kloepper JW (1999) Chitin-mediated changes in bacterial communities of the soil, rhizosphere and within roots of cotton in relation to nematode control. Soil Biol Biochem 31: 551–560.

[9] Piggot PJ, Hilbert DW (2004) Sporulation of *Bacillus subtilis* . Curr Opin Microbiol 7: 579–586. 15556029 10.1016/j.mib.2004.10.001

[10] Tiago I, Teixeira I, Silva S, Chung P, Verissimo A, et al (2004) Metabolic and genetic diversity of mesophilic and thermophilic bacteria isolated from composted municipal sludge on poly-epsilon-caprolactones. Curr Microbiol 49: 407–414. 15696616 10.1007/s00284-004-4353-0

[11] Silo-Suh LA, Stabb EV, Raffel SJ, Handelsman J (1998) Target range of zwittermicin A, an aminopolyol antibiotic from *Bacillus cereus* . Curr Microbiol 37: 6–11. 9625782 10.1007/s002849900328

[12] Borriss R, Chen XA, Rueckert C, Blom J, Becker A, et al (2011) Relationship of *Bacillus amyloliquefaciens* clades associated 1 with strains DSM 7T and FZB42: a proposal for *Bacillus amyloliquefaciens* subsp. *amyloliquefaciens* subsp. nov. and *Bacillus amyloliquefaciens* subsp. *plantarum* subsp. nov. based on their discriminating complete genome sequences. Int J Sys Evol Microbiol 61: 1786–1801. 10.1099/ijs.0.023267-0 20817842

[13] Chen XH, Koumoutsi A, Scholz R, Eisenreich A, Schneider K, et al (2007) Comparative analysis of the complete genome sequence of the plant growth-promoting bacterium *Bacillus amyloliquefaciens* FZB42. Nat Biotechnol 25: 1007–1014. 17704766 10.1038/nbt1325

[14] Koumoutsi A, Chen XH, Henne A, Liesegang H, Hitzeroth G, et al (2004) Structural and functional characterization of gene clusters directing nonribosomal synthesis of bioactive cyclic lipopeptides in *Bacillus amyloliquefaciens* strain FZB42. J Bacteriol 186: 1084–1096. 14762003 10.1128/JB.186.4.1084-1096.2004 PMC344220

[15] Chen XH, Vater J, Piel J, Franke P, Scholz R, et al (2006) Structural and functional characterization of three polyketide synthase gene clusters in *Bacillus amyloliquefaciens* FZB 42. J Bacteriol 188: 4024–4036. 16707694 10.1128/JB.00052-06 PMC1482889

[16] Fan B, Chen XH, Budiharjo A, Bleiss W, Vater J, et al (2011) Efficient colonization of plant roots by the plant growth promoting bacterium *Bacillus amyloliquefaciens* FZB42, engineered to express green fluorescent protein. J Biotech 151: 303–311. 10.1016/j.jbiotec.2010.12.022 21237217

[17] Grosch R, Junge H, Krebs B, Bochow H (1999) Use of *Bacillus subtilis* as a biocontrol agent. III. Influence of *Bacillus subtilis* on fungal root diseases and on yield in soilless culture. J Plant Dis Protec 106: 568–580.

[18] Idriss EE, Bochow H, Ross H, Borriss R (2004) Use of *Bacillus subtilis* as biocontrol agent. VI. Phytohormone-like action of culture filtrates prepared from plant growth-promoting *Bacillus amyloliquefaciens* FZB24, FZB42, FZB45 and *Bacillus subtilis* FZB37. J Plant Dis Protec 111: 583–597.

[19] Yao AV, Bochow H, Karimov S, Boturov U, Sanginboy S, et al (2006) Effect of FZB24 *Bacillus subtilis* as a biofertilizer on cotton yields in field tests. Arch Phytopathol Plant Prot 39: 1–6.

[20] Guel A, Kidoglu F, Tuzel Y, Tuzel IH (2008) Effects of nutrition and *Bacillus amyloliquefaciens* on tomato (*Solarium lycopersicum* L.) growing in perlite. Span J Agric Res 6: 422–429.

[21] Scholz R, Molohon KJ, Nachtigall J, Vater J, Markley AL, et al (2011) Plantazolicin, a Novel Microcin B17/Streptolysin S-Like Natural. Product from *Bacillus amyloliquefaciens* FZB42. J Bact 193: 215–224. 20971906 10.1128/JB.00784-10 PMC3019963

[22] Davis RM., Subbarao KV, Raid RN, Kurtz EA (1997) Compendium of lettuce diseases. APS Press, 15–16pp.

[23] Lugtenberg B, Kamilova F (2009) Plant-growth-promoting rhizobacteria. Ann Rev Microbiol 63: 541–556. 19575558 10.1146/annurev.micro.62.081307.162918

[24] Zhang N, Wu K, He X, Li S, Zhang Z, et al (2011) A new bioorganic fertilizer can effectively control banana wilt by strong colonization with *Bacillus subtilis* N11. Plant Soil 344: 87–97.

[25] Haas D, Defago G (2005) Biological control of soil-borne pathogens by fluorescent pseudomonads. Nat Rev Microbiol 3: 307–319. 15759041 10.1038/nrmicro1129

[26] Raaijmakers JM, Paulitz CT, Steinberg C, Alabouvette C, Moenne-Loccoz Y (2009) The rhizosphere: a playground and battlefield for soilborne pathogens and beneficial microorganisms. Plant Soil 321: 341–361.

[27] Götz M, Gomes NCM, Dratwinski A, Costa R, Berg G, et al (2006) Survival of gfp-tagged antagonistic bacteria in the rhizosphere of tomato plants and their effects on the indigenous bacterial community. FEMS Microbiol Ecol 56: 207–218. 16629751 10.1111/j.1574-6941.2006.00093.x

[28] Grosch R, Dealtry S, Schreiter S, Berg G, Mendoca-Hagler L, et al (2012) Biocontrol of *Rhizoctonia solani*: complex interaction of biocontrol strains, pathogen and indigenous microbial community in the rhizosphere of lettuce shown by molecular methods. Plant Soil 360: 343–357.

[29] Osborne CA, Peoples MB, Janssen PH (2010) Detection of a reproducible, single-member shift in soil bacterial communities exposed to low levels of hydrogen. Appl Environ Microbiol 76: 1471–1479. 20061453 10.1128/AEM.02072-09 PMC2832395

[30] Ling N, Xue C, Huang Q, Yang X, Xu Y, et al (2010) Development of a mode of application of bioorganic fertilizer for improving the biocontrol efficacy to Fusarium wilt. Biocontrol 55: 673–683.

[31] Grosch R, Schneider JHM, Kofoet A (2004) Characterisation of *Rhizoctonia solani* anastomosis groups causing bottom rot in field grown lettuce in Germany. Eur J Plant Path 110: 53–62.

[32] Gutezeit B, Herzog FN, Wenkel KO (1993) Das Beregnungsbedarfssystem für Freilandgemüse. Gemüse 29: 106–108.

[33] Kofoet A, Fricke A, Heine H, Hommes M, Richter E, et al (2001) Kopfsalatsorten und ihre Anfälligkeit gegenüber bodenbürtigen Pathogenen. Gemüse 37: 10–13.

[34] Edwards U, Rogall T, Blöcker H, Emde M, Böttger EC (1989) Isolation and direct complete nucleotide determination of entire genes. Characterization of a gene coding for 16S ribosomal RNA. Nucl Acids Res 17: 7843–7853. 2798131 10.1093/nar/17.19.7843 PMC334891

[35] Lane DJ, Pace B, Olsen GJ, Stahl DA, Sogin ML, et al (1985) Rapid determination of 16S ribosomal RNA sequences for phylogenetic analyses. Proc Natl Acad Sci USA 82: 6955–6959. 2413450 10.1073/pnas.82.20.6955 PMC391288

[36] Osborn AM, Moore ERB, Timmis KN (2000) An evaluation of terminal-restriction fragment length polymorphism (T-RFLP) analysis for the study of microbial community structure and dynamics. Environ Microbiol 2: 39–50. 11243261 10.1046/j.1462-2920.2000.00081.x

[37] Culman SW, Bukowski R, Gauch HG, Cadillo-Quiroz H, Buckley DH (2009) T-REX: Software for the Processing and Analysis of T-RFLP data. BMC Bioinformatics 10: 171. 19500385 10.1186/1471-2105-10-171 PMC2702334

[38] Culman SW, Gauch HG, Blackwood CB, Thies JE (2008) Analysis of TRFLP data using analysis of variance and ordination methods:a comparative study. J Microbiol Meth 75: 55–63. 10.1016/j.mimet.2008.04.011 18584903

[39] Hammer Ø, Harper DAT, Ryan PD (2001) PAST: Paleontological statistics software package for education and data analysis. Palaeontol Electron 4: 9p.

[40] Clarke KR (1993) Non-parametric multivariate analysis of changes in community structure. Aust J Ecol 18: 117–143.

[41] Clarke KR, Gorley RN (2001) Primer v5: User manual/tutorial, Primer-E. Plymouth UK, 91.

[42] Ramette A (2007) Multivariate analyses in microbial ecology. FEMS Microbiol Ecol 62: 142–160. 17892477 10.1111/j.1574-6941.2007.00375.x PMC2121141

[43] Berendsen RL, Pieterse CMJ, Bakker PAHM (2012) The rhizosphere microbiome and plant health. Trends Plant Sci 17: 478–486. 22564542 10.1016/j.tplants.2012.04.001

[44] Nihorimbere V, Cawoy H, Seyer A, Brunelle A, Thonart P, et al (2012) Impact of rhizosphere factors on cyclic lipopeptide signature from the plant beneficial strain *Bacillus amyloliquefaciens* S499. FEMS Microbiol Ecol 79: 176–91. 22029651 10.1111/j.1574-6941.2011.01208.x

[45] Kloepper JW, Ryu CM, Zhang S (2004) Induced systemic resistance and promotion of plant growth by *Bacillus* spp. Phytopathol 94: 1259–1266. 10.1094/PHYTO.2004.94.11.1259 18944464

[46] McCune B, Grace JB (2002) Analysis of ecological communities, MjM Software Design, Gleneden Beach, OR.

[47] Legendre P, Gallagher ED (2001) Ecologically meaningful transformations for ordination of species data. Oecologia 129: 271–280. 28547606 10.1007/s004420100716

[48] Correa OS, Montecchia MS, Berti MF, Ferrari MCF, Pucheu NL, et al (2009) *Bacillus amyloliquefaciens* BNM122, a potential microbial biocontrol agent applied on soybean seeds, causes a minor impact on rhizosphere and soil microbial communities. Appl Soil Ecol 41: 185–194.

[49] Scherwinski K, Grosch R, Berg G (2008) Effect of bacterial antagonists on lettuce: active biocontrol of *Rhizoctonia solani* and negligible, short –term effects on nontarget microorganisms. FEMS Microbiol Ecol 64: 106–116. 18248441 10.1111/j.1574-6941.2007.00421.x

[50] Grosch R, Scherwinski K, Lottmann J, Berg G (2006) Fungal antagonists of the plant pathogen *Rhizoctonia solani*: selection, control efficacy and influence on the indigenous microbial community. Mycol Res 110: 1464–1474. 17127047 10.1016/j.mycres.2006.09.014

[51] Hartmann A, Schmid M, van Tuinen D, Berg G (2009) Plant-driven selection of microbes. Plant Soil 321: 235–257.

[52] Lee B, Lee S, Ryu CM (2012) Foliar aphid feeding recruits rhizosphere bacteria and primes plant immunity against pathogenic and non-pathogenic bacteria in pepper. Ann Bot 110: 281–290. 22437662 10.1093/aob/mcs055 PMC3394643

[53] Yang JW, Yi HS, Kim H, Lee B, Lee S, et al (2011) Whitefly infestation of pepper plants elicits defence responses against bacterial pathogens in leaves and roots and changes the below-ground microflora. J Ecol 99: 46–56.

