# Supplementary material for: Effects of Bacillus amyloliquefaciens FZB42 on Lettuce Growth and Health under Pathogen Pressure and Its Impact on the Rhizosphere Bacterial Community
Source: PLoS One. 2013 Jul 23;8(7):e68818. doi: 10.1371/journal.pone.0068818 (PMC3720850; doi:10.1371/journal.pone.0068818)
Supplement: Table S1 — R test statistic values. (DOCX) [file pone.0068818.s001.docx]

**Table S1.** *R* test statistic values.

|  | **C** | **DYP** | **SYP** | **DYP2x** | **C+*Rs*** | **DYP+*Rs*** | **SYP+*Rs*** | **DYP2x+*Rs*** | **c** | **dyp** | **syp** | **dyp2x** | **c+*Rs*** | **dyp+*Rs*** | **syp+*Rs*** | **dyp2x+*Rs*** |
| --- | --- | --- | --- | --- | --- | --- | --- | --- | --- | --- | --- | --- | --- | --- | --- | --- |
| **C** |  | 0.06218 | 0.0881a | 0.1788 | 0.7688a | 0.9495a | 0.8873a | 0.899a | 0.909a | 0.8485a | 0.7597a | 0.5021a | 0.7824a | 0.8644a | 0.6873a | 0.7922a |
| **DYP** | 0.06218 |  | 0.02915 | 0.1633 | 0.8038a | 0.9824a | 0.9274a | 0.9285a | 0.9218a | 0.8452a | 0.7945a | 0.6158a | 0.8158a | 0.9146a | 0.7236a | 0.8195a |
| **SYP** | 0.08817 | 0.02915 |  | 0.304b | 0.8092a | 0.99a | 0.9146a | 0.948a | 0.9452a | 0.9064a | 0.7859a | 0.5799a | 0.8107a | 0.926a | 0.7024a | 0.8215a |
| **DYP2x** | 0.1788 | 0.1633 | 0.304b |  | 0.8969a | 0.9844a | 0.9391a | 0.9479a | 0.9257a | 0.8386a | 0.9227a | 0.6208a | 0.8699a | 0.9358a | 0.7589a | 0.8984a |
| **C+*Rs*** | 0.7688a | 0.8038a | 0.8092a | 0.8969a |  | 0.07692 | 0.1662 | 0.3073 | 0.9343a | 0.8507a | 0.6798a | 0.4506a | 0.6063a | 0.7785a | 0.5121a | 0.5897a |
| **DYP+*Rs*** | 0.9495a | 0.9824a | 0.99a | 0.9844a | 0.07692 |  | 0.1302 | 0.3552a | 0.9984a | 0.9423a | 0.9406a | 0.6075a | 0.5624a | 0.8814a | 0.5249a | 0.5899a |
| **SYP+*Rs*** | 0.8873a | 0.9274a | 0.9146a | 0.9391a | 0.1662 | 0.1302 |  | 0.3642a | 0.9822a | 0.9554a | 0.8442a | 0.6148a | 0.5978a | 0.8055a | 0.5913a | 0.5641a |
| **DYP2x+*Rs*** | 0.899a | 0.9285a | 0.948a | 0.9479a | 0.3073 | 0.3552 | 0.3642a |  | 0.9369a | 0.9119a | 0.8919a | 0.6235a | 0.5677a | 0.7194a | 0.4842a | 0.6025a |
| **c** | 0.909a | 0.9218a | 0.9452a | 0.9257a | 0.9343a | 0.9984a | 0.9822a | 0.9369a |  | 0.195 | 0.1625 | 0.2304 | 0.5845a | 0.3095 | 0.4298a | 0.5279c |
| **dyp** | 0.8485a | 0.8452a | 0.9064a | 0.8386a | 0.8507a | 0.9423a | 0.9554a | 0.9119a | 0.195 |  | 0.2109 | 0.1857 | 0.6614a | 0.4848a | 0.4653a | 0.5887d |
| **syp** | 0.7597a | 0.7945a | 0.7859a | 0.9227a | 0.6798a | 0.9406a | 0.8442a | 0.8919a | 0.1625 | 0.2109 |  | 0.00125 | 0.616a | 0.2579 | 0.2943d | 0.3484 |
| **dyp2x** | 0.5021a | 0.6158a | 0.5799a | 0.6208a | 0.4506a | 0.6075a | 0.6148a | 0.6235a | 0.2304 | 0.1857 | 0.00125 |  | 0.3468 | 0.2737 | 0.2307 | 0.3028 |
| **c+*Rs*** | 0.7824a | 0.8158a | 0.8107a | 0.8699a | 0.6063a | 0.5624a | 0.5978a | 0.5677a | 0.5845a | 0.6614a | 0.616a | 0.3468 |  | 0.2951 | 0.09128 | 0.1248 |
| **dyp+*Rs*** | 0.8644a | 0.9146a | 0.926a | 0.9358a | 0.7785a | 0.8814a | 0.8055a | 0.7194a | 0.3095 | 0.4848a | 0.2579 | 0.2737 | 0.2951 |  | 0.2124 | 0.3286 |
| **syp+*Rs*** | 0.6873a | 0.7236a | 0.7024a | 0.7589a | 0.5121a | 0.5249a | 0.5913a | 0.4842a | 0.4298a | 0.4653a | 0.2943d | 0.2307 | 0.09128 | 0.2124 |  | 0.1543 |
| **dyp2x+*Rs*** | 0.7922a | 0.8195a | 0.8215a | 0.8984a | 0.5897a | 0.5899a | 0.5641a | 0.6025a | 0.5279c | 0.5887d | 0.3484 | 0.3028 | 0.1248 | 0.3286 | 0.1543 |  |

Values were obtained from analysis of similarity (ANOSIM R: 0.6166; p: 0.0001) between different bacterial communities based on T-RFLP data. The magnitude of *R* indicates the degree of separation between groups of samples, with a score of 1 indicating complete separation and 0 indicating no separation. Letters a,b,c,d indicate different significant Bonferonni- corrected *p* values. C - uninoculated controls, DYP – double young plant treatment one week before planting and four days after planting, SYP – simple young plant treatment two days after planting, DYP2x –young plant treatment before and after planting with double concentration.. +*Rs* – Plants at additional pathogen inoculation of *R. solani*. Capital letters: Sampling after 2 weeks; small letters: Sampling after 5 weeks of growth.
